# Everybody's Page

**Published:** 1888-08-18

**Authors:** 


### EVERYBODY'S PAGE.

It is said that the incandescent electric lamp can be so employed as to produce the hypnotic sleep.

Dr. Russell, the medical officer for the city of Glasgow, reports a group of cases which he defines as epidemic jaundice.

A nervous patient, who thought he had swallowed a pin, telegraphed to his physician asking what he should do, and received by wire the reassuring advice to swallow a pin-cushion.

The University of Bologna, on the occasion of the 800th anniversary of its foundation, conferred its honorary degree upon Sir Spencer Wells, of London, and Dr. S. Weir Mitchell, of Philadelphia.

Dr. Macdougall has noted that purely nervous diseases are relatively rare among the Scotch, a fact which he considers due to their receiving stronger and more stable nerve-centres as gifts of heredity, derived perhaps from a slower speed in the race of life.

According to Dr. Boucheron (*Comptes Rendus*), certain deaf persons do not hear the fundamental sounds—those produced in the larynx—but perceive the harmonic sounds produced in the pharynx, the mouth, or the nose. Dr. Boucheron shows that there are also inverse effects where only the harmonic sounds are heard.

Dr. de Vlaccos expresses the opinion that all substitutes which art can offer for an infant's food in lieu of that intended by nature, have one characteristic in common—that they are all bad, This is like the condemnation frequently lavished on tinned and condensed substitutes for beef tea, of which one grave professor said that they were almost as good for colouring gravies as burnt sugar, but not quite.

Bad teeth are a fertile source of headaches, as they are also of indigestion. Diseased teeth absorb putrefactive substances, which cause irritation of the nervous supply of the teeth, and this leads to pain in the head in much the same way as in the case of the eyesight. The pain is usually felt over the temples or towards the back of the head. It is generally relieved by a purgative followed by a tonic, but the radical cure is the best.

There is a custom of allowing the workmen in the breweries to drink beer rather freely. The men are served with it before they go home to breakfast, before they go home to dinner, before they go home to tea, in addition to another quantum in the middle of the forenoon, and sometimes again in the afternoon. The amount given each time is technically called a "horn," and inquiry regarding the equivalent of this in imperial measure elicits the fact that it is "about a pint." The pint is probably like the Highlander's "mile and a bittock"—an indefinite quantity more than the amount stated; and when it is remembered that the beer served out is what the brewers call "returns," and, further, that it is taken on an empty stomach, it is not surprising that those who have performed the medical duties of dispensaries have had frequent and painful opportunities of studying the evil effects of this baneful system. No one would say hard words about anyone who brews good honest beer, but medical men must protest against those who give a kind of alcoholic hog's wash to their men. Let them run this refuse into its proper place—the gutter; let them, if they like, give their men a glass of decent beer when their work is over for the day; but let them grant slightly higher wages to those who decline to take it; and they will do much to improve the condition of their workmen.

The warm moist climate of Florida has proved particularly beneficial to many forms of phthisis.

It is said that the hardly-expected recovery of the aged Emperor of Brazil from his recent illness was due to the employment of hypodemic injections of caffeine.

The immoderate employment of soap in the washing of the person leads to the soap being imperfectly removed, so that the pores of the skin get clogged up, and healthy skin secretion cannot go on.

Hysteria is a real and most formidable disease, and there is nothing more erroneous than to put down a person suffering from it as a humbug. There is a great tendency, especially among ladies, to utter the expressive word "fiddlesticks" whenever hysteria is mentioned. This, however, is a cruel injustice, which should be protested against.

Professor Scheurlen has announced the discovery of a "cancer bacillus," but as yet the discovery has not been confirmed. One is tempted to fancy that our scientists suffer from a microbe-bacillus mania. A study of our medical journals during the last few years might give an unlearned and nervous man the notion that the world's chief inhabitants are bacteria of various sorts, at whose capricious mercy we poor human creatures live, and move, and have our being.

Any occupation which necessitates severe and continued strain—as in smiths, hammermen, railway workers, &c.—is apt to bring on heart disease. If these men are at the same time working in a close, confined, or heated atmosphere so much the worse, but even in men enjoying an outdoor life it is common as the result of strain—as in shepherds, soldiers, and sailors. In soldiers, the tight-fitting clothes and accoutrements are also blamed as associated with the physical work.

The *Journal des Savants*, in discussing the signs of death, mentions three cases of resuscitation after apparent death. In one of these the body, a general killed, as it was thought, in the retreat from Moscow, had been already buried, but was dug up with the object of removing it to France, and was found to give signs of life. In 1826 a young priest returned to life at the moment when the Bishop of the Diocese was chanting the *De profundis* over his supposed remains.

Queen Elizabeth prefaced her proclamation issued in 1602, prohibiting the erection of new buildings within three miles of London, with the following preamble: "That foreseeing the great and manifold inconveniences and mischiefs which daily grow, and are likely to increase, in the city and suburbs of London, by confluence of people to inhabit the same; not only by reason that such multitudes can hardly be governed, to serve God and obey Her Majesty, without constituting an addition of new officers, and enlarging their authority; but also can hardly be provided of food and other necessaries at a reasonable price; and finally, that as such multitudes of people, many of them poor, who must live by begging or worse means, are heaped up together, and in a sort smothered with many children and servants in one house or small tenement; it must needs follow, if any plague or other universal sickness come among them, that it would presently spread through the whole city and confines, and also into all parts of the realm." The general sanitary improvement of our towns tells first and chiefly upon the children. So also with everything affecting the morals of the adult population. Vice and drunkenness strike at the child through the physical deterioration of the home, and the destruction of that self-denying and scrupulously conscientious discharge of parental responsibility upon which the weak and helpless child is so utterly dependent.

